# PMCA4 (ATP2B4) Mutation in Familial Spastic Paraplegia

**DOI:** 10.1371/journal.pone.0104790

**Published:** 2014-08-13

**Authors:** Miaoxin Li, Philip Wing-Lok Ho, Shirley Yin-Yu Pang, Zero Ho-Man Tse, Michelle Hiu-Wai Kung, Pak-Chung Sham, Shu-Leong Ho

**Affiliations:** 1 Department of Psychiatry, University of Hong Kong, Hong Kong, P. R. China; 2 Centre for Reproduction, Development and Growth, University of Hong Kong, Hong Kong, P. R. China; 3 Centre for Genomic Sciences, University of Hong Kong, Hong Kong, P. R. China; 4 Division of Neurology, Department of Medicine, University of Hong Kong, Hong Kong, P. R. China; 5 Research Centre of Heart, Brain, Hormone and Healthy Aging, University of Hong Kong, Hong Kong, P. R. China; Cambridge University, United Kingdom

## Abstract

Familial spastic paraplegia (FSP) is a heterogeneous group of disorders characterized primarily by progressive lower limb spasticity and weakness. More than 50 disease loci have been described with different modes of inheritance. In this study, we identified a novel missense mutation (c.803G>A, p.R268Q) in the plasma membrane calcium ATPase (*PMCA4, or ATP2B4*) gene in a Chinese family with autosomal dominant FSP using whole-exome sequencing and confirmed with Sanger sequencing. This mutation co-segregated with the phenotype in the six family members studied and is predicted to be pathogenic when multiple deleteriousness predictions were combined. This novel R268Q mutation was not present in over 7,000 subjects in public databases, and over 1,000 Han Chinese in our database. Prediction of potential functional consequence of R268Q mutation on PMCA4 by computational modeling revealed that this mutation is located in protein aggregation-prone segment susceptible to protein misfolding. Analysis for thermodynamic protein stability indicated that this mutation destabilizes the *PMCA4* protein structure with higher folding free energy. As *PMCA4* functions to maintain neuronal calcium homeostasis, our result showed that calcium dysregulation may be associated with the pathogenesis of FSP.

## Introduction

Familial spastic paraplegia (FSP) is a clinically and genetically heterogeneous group of diseases characterized by progressive lower limb spasticity and weakness, and is classified according to phenotype, mode of inheritance and the mutated gene [1]. Pure FSP is characterized by progressive lower limb weakness and spasticity and may be associated with urinary urgency, mild impairment of vibration sense and proprioception. The upper limbs are spared and there is no bulbar dysfunction. Complex FSP is characterized by additional manifestations such as cognitive impairment, epilepsy, cerebellar ataxia, extrapyramidal disturbances, optic atrophy and peripheral neuropathy. Neuroimaging may show white matter lesions, thin corpus callosum, and spinal cord atrophy. FSP can be inherited in an autosomal dominant, autosomal recessive or X-linked fashion [2]. Different mutations in the same gene can cause either pure or complex FSP, and intra-familial phenotypic variability is high, greatly complicating the genetic diagnosis of FSP. Seventy-one forms of FSP (SPG1 to SPG48) have been described involving many gene loci [3], with 20 or more loci associated with autosomal dominant FSP [2]. The associated genes have been reported to be involved in organelle and microtubule dynamics, endoplasmic reticulum homoeostasis, transport, and signal transduction.

In this study, we describe a Chinese family with autosomal dominant FSP. Whole exome sequencing was performed on six family members (4 symptomatic, 2 asymptomatic), and we identified a novel causative mutation, c.803G>A, p.R268Q in the PMCA4 gene.

## Materials and Methods

### Subjects

**Figure 1:**
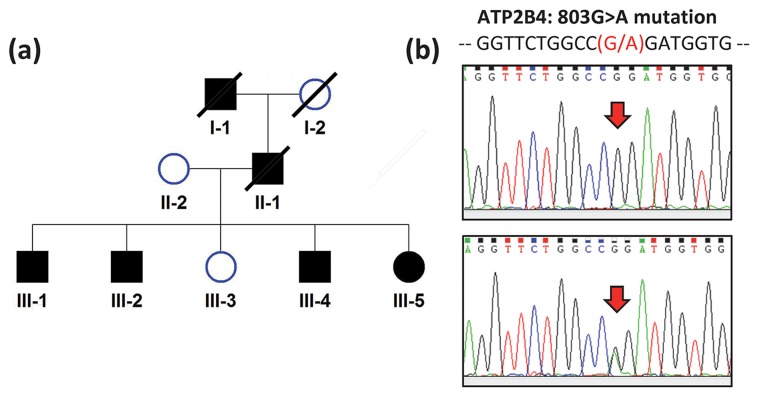
Identification of a Chinese family with autosomal dominant spastic paraplegia. (a) Pedigree. Filled and unfilled symbols indicate affected and unaffected individuals, respectively. Squares and circles represent males and females, respectively. Slashed symbols indicate deceased subjects. (b) DNA sequencing showing PMCA4 (or ATP2B4) R268Q mutation.

Six members of a two-generation Chinese family with FSP were examined (II-2, III-1, III-2, III-3, III-4, III-5) (Fig. 1a). Four were symptomatic and two were asymptomatic. Blood was collected from all six of them and whole exome sequencing was performed.

### Human ethics

This study were reviewed and approved by the Hong Kong Hospital Authority/Hong Kong West Cluster Institutional Review Board Ethics Committee (UW 06-227 T/1252). All subjects gave written informed consent to participate in this study.

### Genomic DNA extraction and exome capture

For gene mutation screening, genomic DNA was purified from peripheral blood leucocytes. Exome capture was conducted by a NimbleGen 2.1 M HD array to enrich for protein-coding regions of human genome DNA (Roche NimbleGen, Inc., Madison, WI, USA). The exon-enriched DNA was sequenced by the Illumina HiSeq 2000 Sequencing platform (Illumina, San Diego, USA) at Axeq Technologies (http://www.axeq.com/).

### Reads mapping and variants calling

The paired-end 101 base-pair (bp) short reads were mapped onto the UCSC human reference genome, version hg19 (corresponding to NCBI Build37), by Burrowa-Wheeler Alignment (BWA) [4]. Duplicated reads were removed by Picard (http://picard.sourceforge.net/). The Genome analysis toolkit (GATK v2.3.9) [5] was used to recalibrate the alignments and to call single nucleotide variants (SNVs) and short insertion-deletion variants (indels). All genotype calls with sequencing read coverage ≤8x, a Phred-scaled mapping calling quality of ≤20, a Phred-scaled base calling quality of ≤50, a Phred-scaled genotype calling quality of ≤20, ≥5% alternative allele supporting reference homozygous genotypes, ≤25% and 70% alternative allele supporting heterozygous and alternative homozygous genotypes, or a Phred-scaled probability of the second possible genotypes ≤50 were excluded.

### Variant filtration and prioritization analysis by KGGSeq

We prioritized the SNVs and Indels by KGGSeq—Knowledge-based mining platform for Genomic and Genetic studies using Sequence data (http://statgenpro.psychiatry.hku.hk/kggseq) [6] (Table 1). First, KGGSeq was used to exclude the following variants sequentially: those with homozygous genotypes in all affected family members and heterozygous in unaffected ones (incompatible with the rare autosomal dominant inheritance of non-consanguineous mating), those with a frequency of over 0.01 in the 1000 Genome Project or dbSNP database or the NHLBI GO Exome Sequencing Project (5600) or our in-house exome sequencing dataset (from over 1,000 Han Chinese), those that do not alter proteins, and those that were predicted to be non-pathogenic based on the deleteriousness scores [7]. We then further prioritized the variants whose gene products have protein-protein interaction (PPI) with the protein of 53 genes causing various types of familial spastic paraplegia and spinocerebellar ataxia: AFG3L2, ATL1, ATN1, ATX1, ATXN1, ATXN10, ATXN2, ATXN3, ATXN7, ATXN8, ATXN8OS, BSCL2, CACNA1A, CYP7B1, FA2H, FGF14, HSPD1, ITPR1, KCNC3, KIAA0196, KIF5A, L1CAM, MJD, NIPA1, PLEKHG4, PLP1, PPP2R2B, PRKCG, REEP1, SLC33A1, SPAST, SPG11, SPG12, SPG14, SPG16, SPG19, SPG20, SPG21, SPG23, SPG25, SPG26, SPG27, SPG29, SPG32, SPG34, SPG37, SPG5B, SPG7, SPG9, SPTBN2, TBP, TTBK2, and ZFYVE26. Similarly, the variants with genes sharing the same biological pathways with some of the 68 genes were highly prioritized as well. Lastly, in a prioritized short list of sequence variants, KGGSeq automatically searches the titles and abstracts of any relevant publications in which the variants' genes and the disease name (familial spastic paraplegia) and two other aliases (hereditary spastic paraplegia and Strumpell-Lorrain disease) were co-mentioned.

**Table 1:** Number of sequence variants after the step-by-step filtration and prioritization in KGGSeq.

Steps	# SNVs (Genes)	#Indels (Genes)
Initial	91,469	34,308
Inheritance pattern^1^	855	87
Rare in dbSNP+1000 Genome+ESP and an in-house dataset^2^	64	31
Protein altering variants^3^	9	1
Predicted to be pathogenic	6(6)	–
**Knowledge-related^4^**		
PPI	1 (1)	–
Pathway	3(3)	–
PubMed	0	–

**Notes**: 1: Dominant mode only considered with variants in heterozygous genotypes and with shared alleles between the two patients; 2: The rare variants referred to variants with MAF≤1% in the datasets; 3: This category includes missense, stopgain, stoploss and splicing single nucleotide variants and insertions/deletions causing frameshift, nonframeshift, stoploss, stopgain and splicing differences; 4: Knowledge-related variants/genes refer to those variants' genes having PPI(s) or sharing pathway(s) with at least one known causal gene of FSP and those variants fell into gene(s) which were co-mentioned in the titles or abstracts of papers in the PubMed database.

We also carefully screened our patients for non-synonymous mutations among 61 FSP candidate genes [including 47 genes causing various types of Familial Spastic Paraplegias (AFG3L2, ALS2, AP4B1, AP4E1, AP4M1, AP4S1, AP5Z1, ATL1, BSCL2, C12orf65, CCT5, CYP2U1, CYP7B1, DDHD1, ELOVL4, ERLIN2, FA2H, GAD1, GJA1, GJC2, HSPD1, KANK1, KIAA0196, KIF1A, KIF5A, L1CAM, NIPA1, PLP1, PNPLA6, REEP1, RTN2, SLC16A2, SLC33A1, SPAST, SPG11, SPG20, SPG21, SPG7, TECPR2, VCP, VPS37A, ZFYVE26, B4GALNT1, C19orf12, GBA2, NT5C2 and ZFYVE27) [3], [8] and 14 newly proposed genes (ARL6IP1, ERLIN1, KIF1C, USP8, WDR48, AMPD2, ENTPD1, ARSI, DDHD2, PGAP1, FLRT1, RAB3GAP2, MARS and ZFR) by Novarino et al. [3]. Finally, we replicated the short list of sequence variants by conventional Sanger sequencing in all available family members to exclude false positives of the high-throughput sequencing.

### Computational modeling

AGGRESCAN (http://bioinf.uab.es/aggrescan) was used to evaluate the contribution of the mutation to protein folding properties. The protein tertiary (or 3D) structures was built by SWISS-MODEL (http://swissmodel.expasy.org/) based on the data from PDB website (http://www.rcsb.org/pdb/home/home.do). PyMOL (http://www.pymol.org/) was used to render tertiary structure of proteins and to predict the potential functional consequence of a missense mutation on a protein. The iPBA (http://www.dsimb.inserm.fr/dsimb_tools/ipba/) [9] was used to compare the structure differences between the wild type and mutant proteins. PopMuSic-2.0 (http://babylone.ulb.ac.be/PoPV2) was used to predict thermodynamic protein stability changes based on the built protein tertiary structure [10].

## Results

### Clinical examination of the FSP family

Six members of a two-generation Chinese family with FSP were clinically examined (II-2, III-1, III-2, III-3, III-4, III-5) (Fig. 1a). Four were symptomatic and two were asymptomatic. Proband (III-1) presented at 44 years with progressive spastic paraplegia since mid-30's (Fig. 1a). He had brisk lower limb tendon reflexes and bilateral ankle clonus but downgoing plantar responses. There was bilateral lower limb spasticity but no dystonia or other parkinsonian features. Reassessment ten years later revealed increased spasticity with mild deterioration in muscle strength (4/5) and sparing of muscle bulk. He remained ambulatory with spastic gait. His upper limbs remained unaffected, without cognitive, cerebellar or bulbar involvement. His late paternal grandfather and father had similar features. III-2 developed progressive spastic paraplegia from teenage. Examination at age 41 years showed mild weakness in hip and knee flexion, with brisk knee and ankle reflexes and downgoing plantar responses bilaterally. He has slow progression over the next 9 years but remained ambulatory despite occasional falls. He has no upper limb, cerebellar, bulbar, cognitive or extrapyramidal involvement. III-4 has abnormal gait since teenage, and has difficulty running. Examination showed weakness in hip flexion with mild hypertonia, brisk lower limb tendon reflexes and ankle clonus bilaterally. III-5 had a similar presentation as III-4 when she was assessed at age 34 years. III-3 remained non-symptomatic with normal neurological examination when assessed at age 39 years and at reassessment 10 years later. In the affected subjects, neuroimaging including MRI brain and spine did not reveal any clinically relevant lesions. Whereas all the symptomatic family members developed definite physical signs of FSP by their 30's, II-2, the mother of the affected patients, was asymptomatic with normal neurological examination at age of 70 years. III-3 did not complain of any symptoms and had normal neurological examination findings at age 49 years.

### Exome sequencing and identification of candidate genes

Exome sequencing was performed on four affected family members (III-1, III-2, III-4 and III-5) and two unaffected members (II-2 and III-3). We first screened for non-synonymous mutations in the 61 candidate genes of FSP. About 99.53% of the coding regions of these 61 candidate genes were covered by the NimbleGen 2.1M HD capture we used. According to RefGene the total length of these unique coding regions is 141,752 bp and around 94–95% of these sequences had the minimum coverage 4X in each of the 6 sequenced subjects. We observed 24 non-synonymous SNVs in 19 of these genes. However, none of them co-segregated with the phenotype in the family members. No frame-shift or non-frame-shift indel mutations were found in any of these genes. These results suggested that the disorder our patients suffer from may be caused by a mutation in a gene that had not been described in FSP before.

Initially, there were 982,710 SNVs and 34,308 indels called from the aligned short reads by GATK. After stringent quality control on KGGSeq (see criteria in Materials and Methods section), 61,542 SNVs and 4,715 indels were retained (Table 1). Around 99% of SNVs and Indels were inconsistent with the dominant inheritance mode and eliminated. After exclusion of variants that do not alter protein, were non-rare (MAF>0.01) and predicted to be non-pathogenic, only 6 SNVs of different genes remained (Table 1).

Among the 6 probable pathogenic SNVs (Table 2), the missense mutation, c. 803G>A, of PMCA4 gene had the highest pathogenic prediction probability [7]. All four symptomatic patients have the mutant allele A. The two asymptomatic family members, and over 1,000 Chinese subjects in our internal database, and other over 7,000 subjects in the public reference databases do not have this mutant allele. This missense mutation resulted in an amino acid substitution at the same site, p.R268Q, of both protein isoforms, NP_001001396.1 and NP_001675.3 (Fig. 1b). In our patients, we observed that this mutation was surrounded by 4,753 consecutive sequence variants (covering ∼61million base-pair) with identity-by-state allele over 1, suggesting a long region shared by our patients. Furthermore, the protein product of PMCA4 gene had indirect protein-protein interaction (PPI) and shared the same biological pathways with some of the 68 known FSP and spinocerebellar ataxias causal genes. In the PubMed database, no publication simultaneously mentioning the short listed genes and the disease name or aliases in the title or abstract was found.

**Table 2:** The 6 predicted pathogenic in the prioritized short list.

Chr.	Pos.	Ref./Alt.^1^	Symbol	Max Alt. AF^2^	PPI	Shared Pathway^3^	IBS Region Length (bp)	Pathogenic Prob.^4^
**1**	203669953	G/A	ATP2B4	NA	ATP2B4<->DLG1<->SPG21, ATP2B4<->DNAH8<->HSPD1, DNAH8<->SPAST, DNAH8<->AFG3L2	KEGG_CALCIUM_SIGNALING_PATHWAY#178: (ATP2B4, PRKCG, ITPR1, CACNA1A); DAVICIONI_TARGETS_OF_PAX_FOXO1_FUSIONS_DN#68: (ATP2B4, REEP1, ITPR1);	61,634,260	0.145
**1**	232626709	C/T	SIPA1L2	0.000119		HORIUCHI_WTAP_TARGETS_UP#306: (SIPA1L2, ATL1, ITPR1); WANG_SMARCE1_TARGETS_UP#280: (SIPA1L2, PLP1, ATXN1)	61,634,260	0.107
**12**	8202068	G/A	FOXJ2	0.0009			426,500	0.043
**12**	9353933	A/G	PZP	0.0005			2,660,223	0.039
**14**	47426686	C/G	MDGA2	0.0037			11,708,122	0.039
**21**	43242351	C/T	PRDM15	NA			4,343,771	0.039

**Notes**: 1: Reference allele and alternative allele; 2: The maximal frequency of the alternative allele in one of reference datasets; 3: The 4850 curated gene sets from Curated gene sets from MSigDB 3.1 (http://www.broadinstitute.org/gsea/msigdb/genesets.jsp?collection=C2) were used; 4: It is a posterior probability given their deleteriousness and functional scores and the prior probability 0.05.

The co-segregation of the missense mutation at PMCA4 with disease status in all 6 family members was confirmed by Sanger sequencing.

### Computational modeling of the mutant PMCA4 protein

**Figure 2:**
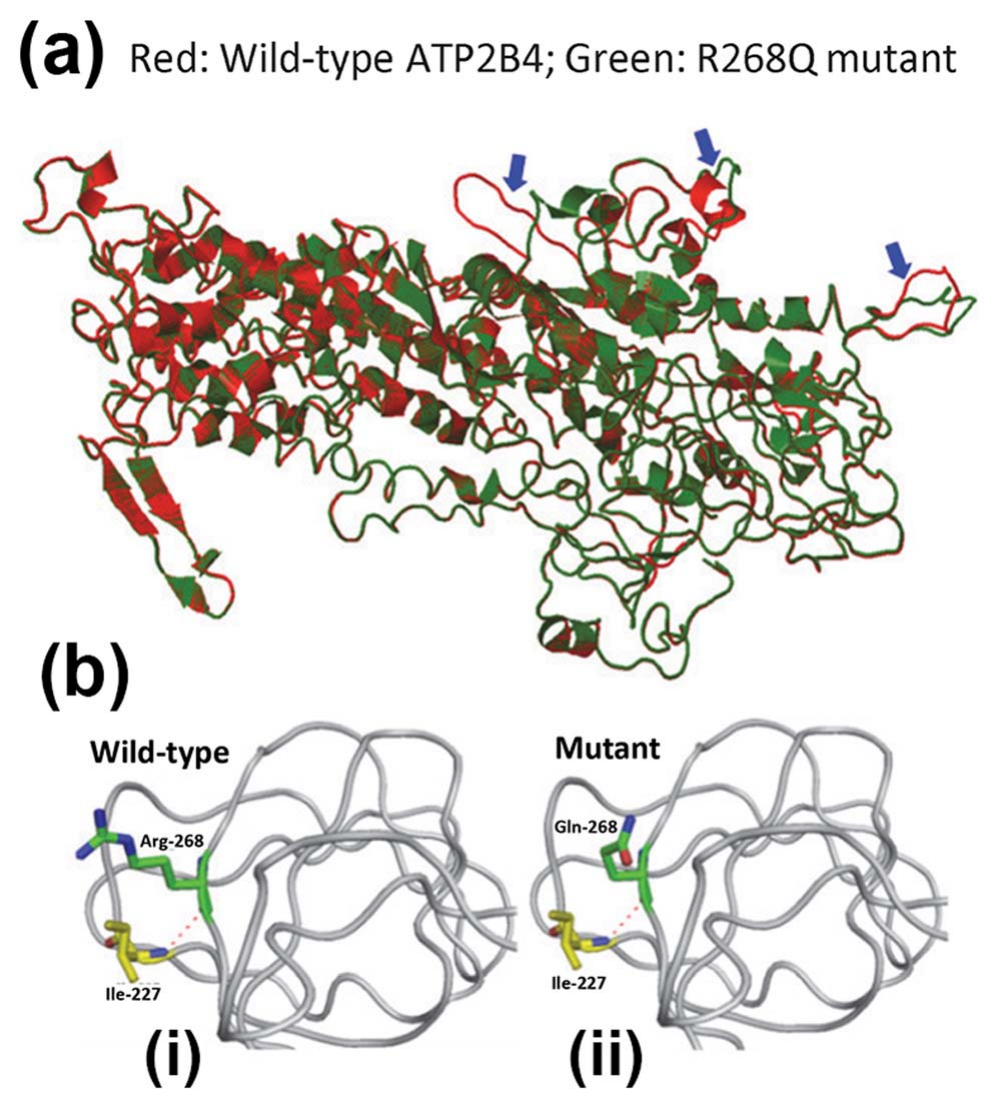
Computational modeling of R268Q ATP2B4 mutant protein. (a) Local tertiary molecular structure of, (b–i) wild-type and (b–ii) mutant PMCA4 (or ATP2B4) protein. The red dashed line denotes hydrogen bond.

We evaluated the impact of the mutation, c. 803G>A (p.R268Q), on *PMCA4* protein structure (Fig. 2a,b). AGGRESCAN was used to evaluate the potential effects of the mutation on protein folding properties. PyMOL was used to render protein tertiary structure and to predict the potential functional consequence of a missense mutation on the protein. According to AGGRESCAN, p.R268Q is located in protein aggregation-prone segment suggesting that it may cause protein misfolding [11]. As there is no experimental tertiary structure information on *PMCA4* proteins, we used the SWISS-MODEL based on the data from PDB website to predict the 3D structures of wild-type and mutant *PMCA4* proteins [12]. Analysis with PopMuSic indicated that this mutation would cause higher folding free energy (ΔG = 0.02 kcal/mol) that may destabilize the *PMCA4* protein structure [10]. The uncharged residue (Glutamine, Q) in mutant protein has a different configuration from the positively charged residue (Arginine, R) in wild-type protein (Fig. 2a). Structure alignment analysis by iPBA found 3 local differences in the 3D structure between wild-type and mutant proteins [Fig. 2b (i–ii)], which may affect protein function [13].

## Discussion

*PMCA4* belongs to the family of plasma membrane Ca^2+^-ATPases consisting of 4 isoforms with dozens of variants generated by alternative RNA splicing [14]. Although mutations in *PMCA2* and *PMCA3* have been reported in congenital hearing loss and X-linked cerebellar ataxia respectively [15], mutations in *PMCA4* have not hitherto been associated with other human disease. The PMCA4 gene is known to have variable sequence. There are 102 non-synonymous SNVs observed in the 1,000 Genomes Project [16] and ESP [Exome Variant Server, NHLBI GO Exome Sequencing Project (ESP), Seattle, WA (URL: http://evs.gs.washington.edu/EVS/)]. However, only 8 variants had alternative allele frequencies ranging from 1% to 3%. All the other variants were rare. The p.R268Q variant that was identified in the symptomatic members of the family was not found in any of the public databases, or in our internal database. There was perfect co-segregation of the p.R268Q mutation with disease status in our family. This mutation is predicted to be pathogenic when combining multiple deleteriousness predictions [including SIFT [17], Polyphen2 [18] and MutationTaster [19]. PMCA4 has protein-protein interaction and shares the same pathways with some known causal genes of FSP and spinocerebellar ataxias.

*PMCA4* is expressed ubiquitously in the adult but is the only isoform which is localized in lipid rafts in pig cerebellum [20]. Lipid rafts exist in neuronal dendrites where postsynaptic protein complexes are localized. Thus, *PMCA4* may play a role in signaling pathways at synaptic nerve terminals, where the synaptic activity is highly dependent on calcium signaling [21]. Dysregulation of calcium signaling in brain is commonly associated with various neurodegenerative diseases, e.g. Alzheimer's disease, Parkinson's disease, and amyotrophic lateral sclerosis [22]. The PMCA4 R268Q mutation may be pathologically important because the potential deficiency in removing cytosolic free calcium may cause transient accumulation of free Ca^2+^ (calcium overload) between neuronal excitation, and may result in subsequent activation of various cell death pathways, e.g. Ca^2+^-dependent synthases and proteases to damage cytoskeleton, membrane, and DNA leading to excitotoxicity and neuronal death [23]. Taken together, we postulate that the p.R268Q mutation in PMCA4 identified in this family caused neuronal deficits associated with FSP. This is the first report to demonstrate *PMCA4* mutation to be associated with autosomal dominant FSP, indicating that calcium dysregulation may be involved in the pathogenesis of spastic paraplegia. The detailed pathogenic mechanism of how impairment in neuronal calcium flux can directly cause the disease phenotype in FSP requires further studies.
